# Innovative Passive and Environmentally Friendly System for Improving the Energy Performance of Buildings

**DOI:** 10.3390/ma15207224

**Published:** 2022-10-17

**Authors:** Andrei Burlacu, Gavril Sosoi, Chérifa Abid, Marinela Barbuta, Marina Verdes, Robert Stefan Vizitiu, Marius Branoaea

**Affiliations:** 1 Faculty of Civil Engineering and Building Services, “Gheorghe Asachi” Technical University of Iasi, 700050 Iasi, Romania; 2 Aix-Marseille Université, CNRS, IUSTI, 13453 Marseille, France

**Keywords:** energy efficiency, passive system, solar heat flux, heat pipes

## Abstract

The aim of the study is to develop a system for converting, accumulating, and delivering solar energy that is based on the development of an innovative solar panel with heat pipes and a heat storage wall, for the construction of passive structures. The novel aspect of this experiment is the utilization of concrete walls that have different recyclable materials added to their structure in various proportions. The solar energy from the sunny façades is transformed by this system into thermal energy, which is then transferred by integrated heat pipes in a massive element with high thermal inertia. Using insulated shutters, thermal energy can be stored during the day and released at night to keep the room at a comfortable temperature. In order to integrate the modules into the solar recovery system, four concrete samples were cast with a blend of standard and waste aggregates. Four heat fluxes of 100 W/m^2^, 150 W/m^2^, 200 W/m^2^, and 250 W/m^2^ were applied to each global system. Thermal imaging data and numerical simulations both supported the findings of temperature sensors. The most effective mixture, fly ash and chopped PET, delivered temperatures that were, on average, 3.3% higher at the end of the charging cycle than those measured for the control sample. The discharging cycle of the concrete block with fly ash and sawdust was the most effective, with an average temperature loss of 5.0 °C as compared to 5.5 °C for the control sample, on average.

## 1. Introduction

Energy efficiency in buildings is a constant challenge for researchers, who must come up with new solutions to reduce energy consumption. New technologies proposed and developed for energy performance in buildings must take into account two criteria: low cost of production and environmentally-friendly manufacturing.

Energy performance in buildings became a priority in recent decades, and the use of passive systems represents a great alternative due to their capability to convert or to store heat received from solar radiation. The use of green walls is one of the passive methods for lowering energy use in buildings. Their effects have been researched in both warm and cold climates, including oceanic [1], temperate [2], and subtropical [3]. These passive energy-saving solutions have also been categorized by Perez et al. [4]. Louis et al. [5] examined how green walls affect the thermal characteristics of the building envelope and how solar radiation absorption, reflection, and transmissivity are impacted.

Trombe walls are also used to make buildings more energy efficient. They have also been researched in a variety of climates, including the desert regions of Iran [6], the cold regions of Turkey [7], and the city of Athens, Greece [8]. Various studies have examined the possibility of improving Trombe walls by analyzing the conditions of ventilation openings and occlusion device operation [9], or by creating innovative designs [10].

Buildings can also save energy by using walls that include phase-change materials. Researchers have looked into a number of ways to introduce PCM into the wall structure. In order to preserve similar comfort levels throughout the year, Faraji et al. [11] quantitatively analyzed the feasibility of replacing the thick and heavy external walls utilized in Mediterranean nations with thin and light thermal mass walls. The findings demonstrate that internal temperature variations in the wall using PCM are significantly reduced. Omari et al. [12] examined how the addition of micro-dispersed PCM-composite boards alters a wall’s thermal behavior throughout the year.

In addition, Meng et al. [13] and Xie et al. [14] examined the behavior of rooms with PCM-composite walls throughout the year. Chen et al. [15] examined another usage of PCM walls by putting them in solar greenhouses. The suggested approach improved the wall’s ability to store and release heat, raising the average soil temperature, indoor air temperature, and daily effective accumulative temperature, on average.

The use of double-skin façades is another passive energy-saving technology. Studies like those by Dama et al. [16] and Souza et al. [17] use experimental and numerical analysis to examine how introducing a double-skin façade to a building influences heat gains inside the building. Zhang et al. [18] attempted to improve the DFS by figuring out the ideal vertical air channel thickness. They discovered that the thickness should be less than 0.6 m for air ducts intended to boost ventilation and less than 0.2 m for those intended to increase warm air supply. Amorphous silicon photovoltaic blinds may be used to generate power on-site while also reducing heat absorption or loss through glazing, according to research by Luo et al. [19]. To lessen the additional rise in cooling loads in buildings, Li et al. [20] inserted a PCM into a double-skin façade.

Another solution for improving the performance of passive systems is to integrate heat pipes in the wall structure. A study by Zhang et al. [21] proposed this type of passive solar energy utilization technology, where the heat transfer and energy-saving characteristics of the wall were analyzed theoretically and experimentally. In a similar manner, Tan et al. [22] investigated the heat transfer and energy-saving capabilities of wall-implanted heat pipes during the heating season.

The heat transfer performance of the proposed systems was validated by conducting the average equivalent heat transfer coefficient experiment. Heat pipe evaporation section length and diameter were also optimized to calculate the energy-saving potential. They reported that the proposed system wall, implanted with heat pipes, has remarkable energy-saving potential. Liu et al. [23] analyzed the possibility of improving the structure of heat pipes implanted in walls, for a better heat transfer performance.

Li et al. [24] and Zhang et al. [25] investigated the dynamic heat transfer performance of the wall implanted with heat pipes during heating season. According to their report, the suggested method can save 21.61% of energy, and the heat flow ratio to the inside of the wall is 4:1. In the same manner, the wall with heat pipes implanted was analyzed during the winter season by Sun et al. [26].

The performance of a proposed heat pipe-assisted solar wall was investigated by Michael et al. [27] through simulation and experiments. The heat pipe model was calculated using electrical resistances, and to simulate hourly performance, MatLab codes were used. The investigation of the heat pipe-assisted solar wall was continued by Brian et al. [28] who, based on the numerical and experimental results, proposed a full-scale model to analyze the performance during the heating season. Continuing the research on the subject, Brian et al. [29] proposed the use of the solar heat pipe system to reduce thermal gains in the cold season.

In this study, we evaluated a passive heating system that harnesses solar energy and converts it into thermal energy through the use of heat pipes inserted in walls. The novel aspect of this experiment is the utilization of concrete walls that have different recyclable materials added to their structure in various proportions.

## 2. Materials and Methods

### 2.1. Description of the Global System

**Figure 1:**
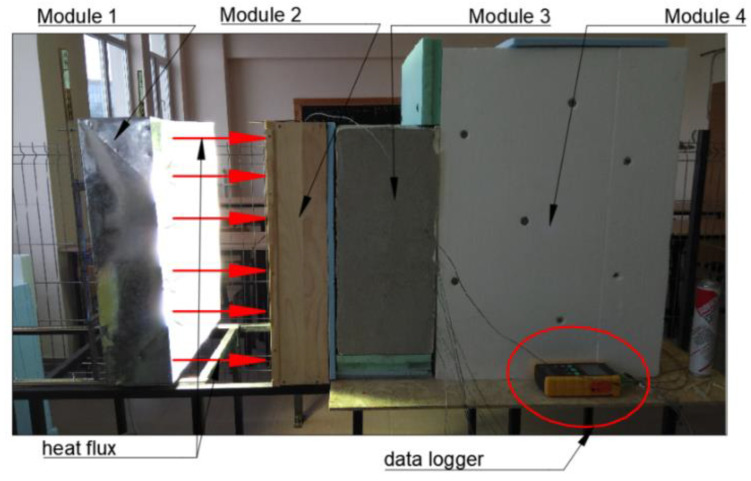
The experimental setup of the global system.

The newly-designed passive system was built in four modules (Figure 1). Module one consists of a solar radiation simulator built using six lamps that are arranged in two columns with 3 lamps in each column. The second module consists of a wood shell with a height of 0.8 m, a width of 0.6 m, and a thickness of 0.15 m, called the air gap. A layer of high-density polystyrene is applied inside the shell. The air gap has a glass layer in front and a black painted steel plate at the back. The glass layer is 0.05 m thick and was placed to allow the solar radiation to pass through and reach the absorber plate.

The third module consists of an insulated concrete wall equipped with heat pipes. The concrete block has a length of 0.6 m, a width of 0.4 m, and a thickness of 0.2 m. Four concrete samples were cast with standard aggregates and waste aggregates in the mixture: CW01—corresponds to the concrete control sample; CW02—corresponds to concrete with 10% fly ash replacement for cement and 20% chopped PET replacement for the sand mass; CW03—corresponds to concrete with 10% fly ash replacement for cement and 20% sawdust replacement for the sand mass; CW04—corresponds to concrete with 10% fly ash replacement for cement, 10% granular polystyrene with dimensions between 1 and 4 mm replacement for the sand mass and 20% granular polystyrene with dimensions between 4 and 8 mm replacement for the aggregate sort. The 4th module consists of the insulated room built from wood plates. This module is insulated using a polystyrene layer of 0.1 m at the exterior to prevent heat loss. The concrete blocks were built according to previous research [30], which analyzed the density, compressive strength, and thermal conductivity of several concrete samples mixed with various percentages of fly ash, chopped PET, sawdust, and granular polystyrene.

### 2.2. Experimental Approach to the Global System

The designed model was subjected to experimental investigations in the laboratory. The air temperature inside the laboratory measured during the experimental investigations was 15 °C. The solar radiation simulator was used to replicate the heat flux emitted by solar radiation. The heat fluxes proposed for the experimental investigations of the heat pipe-equipped concrete walls are 100 W/m^2^, 150 W/m^2^, 200 W/m^2^, and 250 W/m^2^.

**Figure 2:**
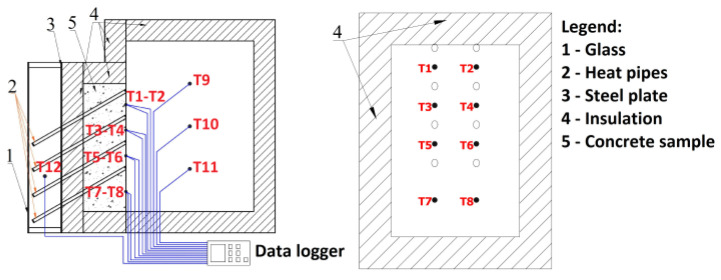
Experimental setup and thermocouples position.

The discharge of the heat captured by the heat pipe was measured on the external surface of the wall inside module 4 and in the center of module 4. For both systems, constant heat flux was applied to the glass side of the solar collector for 10 h, which was considered the charging time. At the end of the charging cycle, the solar module was turned off and the discharging cycle began, continuing for the next 14 h. Temperature measurements were made with type K thermocouples and recorded by a data logger on a memory card. There were 8 sensors on the surface of the wall, and 3 sensors in the middle of module 4, placed at different heights. The data logger recorded the temperatures every 60 s during the charging and discharging cycle. The placement of the sensors are presented in Figure 2.

### 2.3. Numerical Approach to the Global System

**Figure 3:**
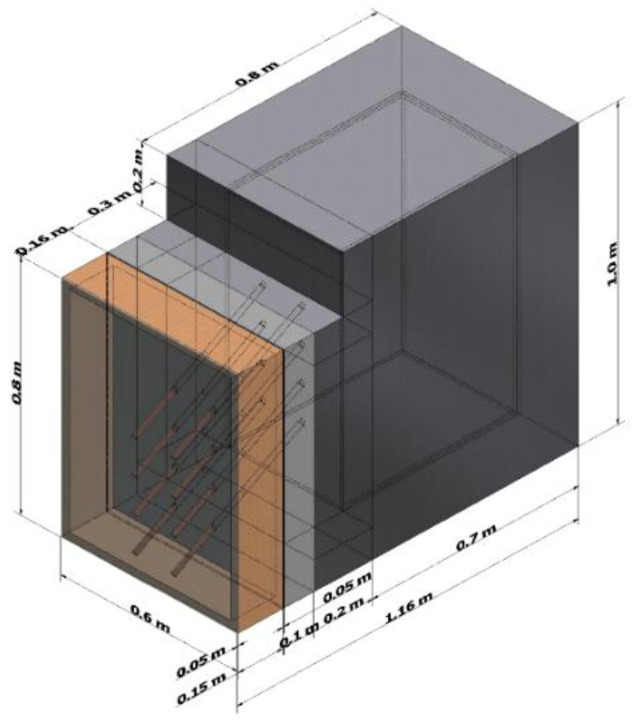
The 3D design of the global system.

To choose an optimum design for the experimental stand, the global system was designed in 3D using Autodesk Inventor software and subjected to numerical simulations using Autodesk CFD software. The geometry of the global system presented in Figure 3 was reproduced at a real scale for the modules built in the experimental model.

The geometry was imported in Autodesk CFD, suppressing the components with no use in the global system to reduce the computational time required to reach the convergence. The simulations ran based on the governing equations of fluid dynamics or Navier-Stokes equations: the continuity, momentum, and energy equation coupled to the energy conservation equation. The software resolved the conjugate heat transfer where conduction, convection, and radiation heat transfer are coupled with fluid flow.

A constant temperature of 15 °C was applied to the exterior air volume, which reproduced the initial conditions of laboratory indoor air. Adiabatic conditions were imposed for the insulated components, leading to a heat flux of φ = 0 W/ m^2^ applied on the polystyrene surfaces. Various boundary conditions of heat fluxes, from 100 to 250 W/m^2^, were applied on the absorber plate and heat pipe evaporator surfaces. Due to the software’s limitation with regard to simulating the heat pipe multiphase change when the evaporation-condensation process occurs, a new material with a very high and constant thermal conductivity was created and assigned to the heat pipe inner volume. From the software database, materials were assigned: glass for the glass; wood for the wood shell of the solar heat absorber and for the room; and copper for the heat pipes. The geometry was discretized in 300,000 elements using the mesh generator of the software. Based on automatic local curvature, the meshing of the solid and fluid zones was generated at a medium size while the contact between them was generated at a smaller size for better capture of the heat transfer. Assuming a laminar incompressible flow at hydrostatic pressure, the heat transfer mechanisms by radiation, convection, and conduction were proposed to be resolved during the simulation.

## 3. Results

### 3.1. Experimental Results

#### 3.1.1. Charging Cycle

All 4 concrete samples were subjected to a 10 h charging cycle and a 14 h discharging cycle. The data logger recorded the temperatures for all 11 sensors every 60 s. Table 1, Table 2, Table 3 and Table 4 present the average temperature at the surface of the concrete blocks recorded by the sensors at specific moments, after one hour, five hours, and at the end of the charging cycle (10 h).

#### 3.1.2. Discharging Cycle

**Figure 4:**
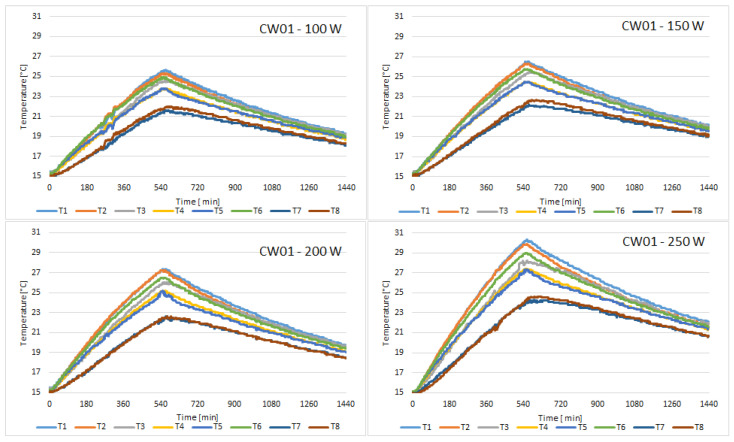
Temperature variation recorded by the sensors for concrete sample CW01.

The discharging cycle begins once the solar simulator is turned off, after 10 h. The cycle lasts for 14 h, thus completing a 24 h cycle. The following figures present the temperature variation of all 8 sensors placed on the external face of the concrete wall, for all of the samples tested. Figure 4 presents the variation of temperature recorded by the sensors for the control sample.

In the first hour, the heat pipes increased the average temperature of concrete sample CW01 by 1 °C in all 4 tests. We can observe that when the heat flux is 250 W/m^2^, the temperature rise is the fastest. The temperature peak inside the room is 24.7 °C at the end of the charging cycle.

**Figure 5:**
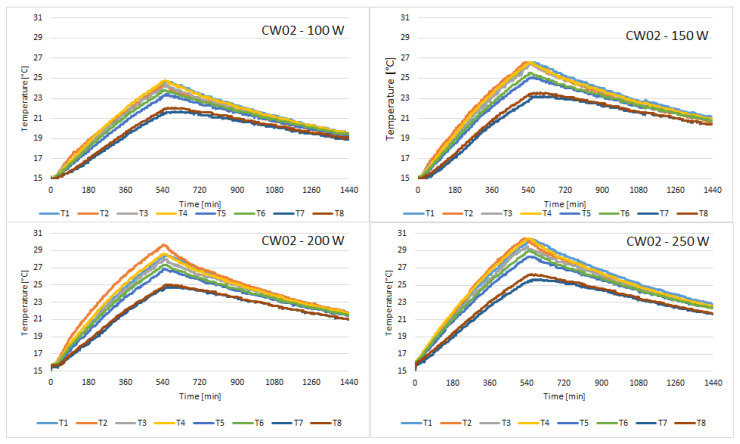
Temperature variation recorded by the sensors for concrete sample CW02.

Compared to CW01, the temperature on the face of CW02 sample is closer to uniformity on the 8 sensors. It can be observed in Figure 5 that the temperature difference between the T2 sensor which is placed in the upper zone and the T7 sensor which is placed in the lower zone of the concrete block is smaller for CW02 than for CW01. This means the thermal conductivity of this concrete with fly ash and chopped PET is superior to simple concrete.

**Figure 6:**
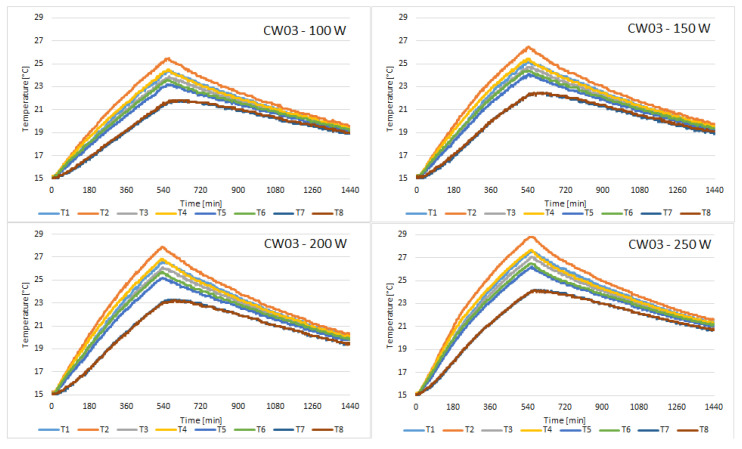
Temperature variation recorded by the sensors for concrete sample CW03.

Probe CW03 revealed the most uniform temperature distribution, but the peak temperatures were smaller than the temperatures recorded for CW01. With the highest heat flux, the temperature of sensor T1 barely reached 29 °C at the end of the charging cycle, as can be observed in Figure 6.

**Figure 7:**
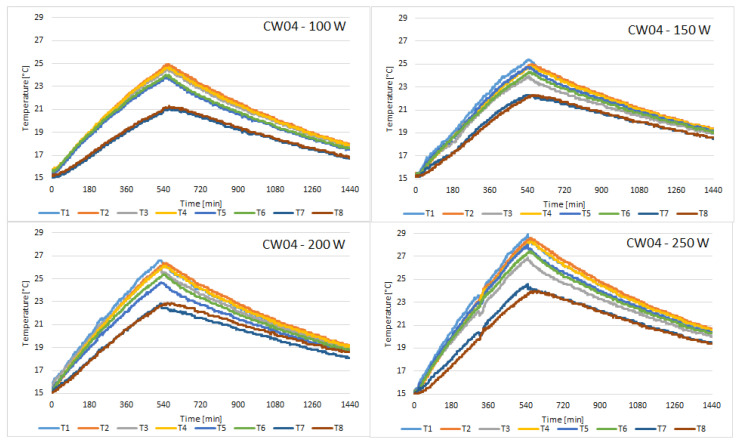
Temperature variation recorded by the sensors for concrete sample CW04.

The temperatures recorded for probe CW04 were very similar to those recorded for probe CW03. Figure 7 presents the variation of temperature recorded by the sensors.

With the temperature values recorded by the sensor, the conductive heat transfer was calculated at the end of the charging cycle and at the end of the discharging cycle using Equation (1), constituting the heat gain of the wall.

(1) $$Q=\frac{k\times A\times ∆T}{d}\;\left[W\right]$$

**Figure 8:**
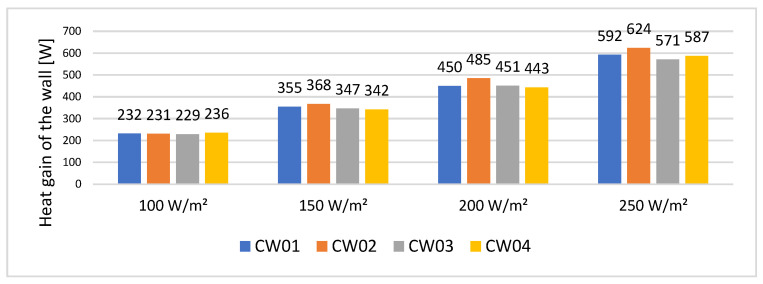
Heat gain of the wall.

When the imposed heat flux is at 100 W/m^2^, all 4 concrete samples behave similarly; however, when the heat flux is increased we can observe that CW02 has the highest heat gain (Figure 8).

Considering that the area of the wall is 0.24 m^2^, the heat flux through the wall is presented in Table 5.

#### 3.1.3. Infrared Camera Measurements

**Figure 9:**
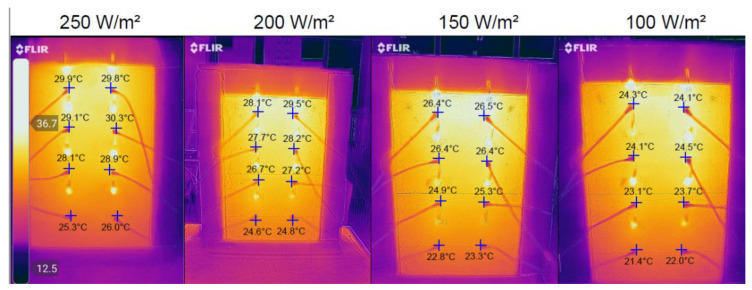
Infrared camera photos for CW02.

At the end of the charging cycle, each concrete block was photographed using a FLIR thermal camera. The sensors of the camera were set on the temperature thermocouples to compare the values recorded by the sensors to the values recorded by the infrared camera. Photos of the CW02 concrete block were chosen as an example since they represent the highest temperatures reached by a concrete block during this experiment. The results can be observed in Figure 9.

Table 6 presents the comparison between the temperatures recorded by the sensors and the temperatures recorded by the infrared camera. Since the accuracy of the camera is ±5 °C, the results were slightly smaller than the experimental results, by 0.1 to 0.3 °C, which are within the admissible measurement error range. The rest of the experiments had similar results.

### 3.2. Results of the Numerical Simulations

**Figure 10:**
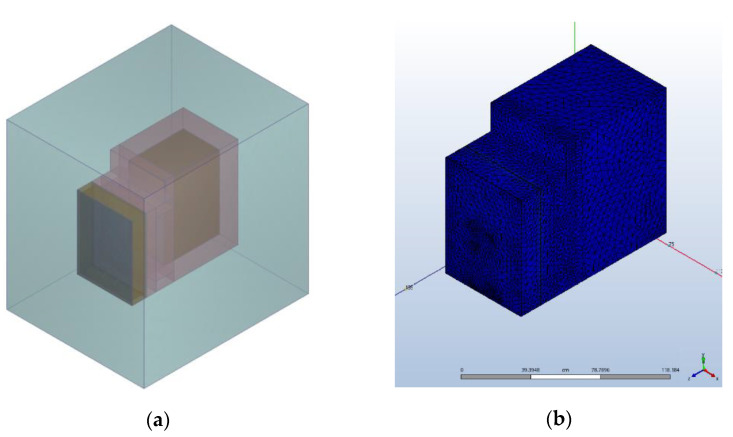
The global system imported in the simulation environment: (**a**) the global system inside the air volume; (**b**) meshing of the global system.

The numerical simulations were focused on the charging cycle. The global system geometry was designed in Autodesk Inventor, reproducing the components built in the experimental model at a real scale. In order to speed up the convergence process, the geometry was loaded into Autodesk CFD while the thermal transfer-related components were suppressed. Around the global system, an air volume of 4.9 m^3^ was generated to reproduce the laboratory conditions. Using the automatic mesh generator, the geometry was discretized, resulting in 300,000 elements. Figure 10a,b present the geometry imported in the CFD Simulation environment, and the meshing applied to the 3D model, respectively. Based on automatic local curvature, the meshing of the solid and fluid zones was generated at a medium size while the contact between them was generated at a smaller size to better capture the heat transfer. Assuming a laminar incompressible flow at hydrostatic pressure, the heat transfer mechanisms by radiation, convection, and conduction were proposed to be resolved during the simulation.

All four concrete blocks were created in the CFD simulation environment and were assigned a custom material with their specific thermal properties. To see the influence of heat pipes inserted inside the concrete block, the first 3D model was created without heat pipes inserted and was subjected to a heat flux of 250 W/m^2^. It was named CW00. The comparison is presented in Table 7. The influence of heat pipes can be observed for every block.

**Figure 11:**
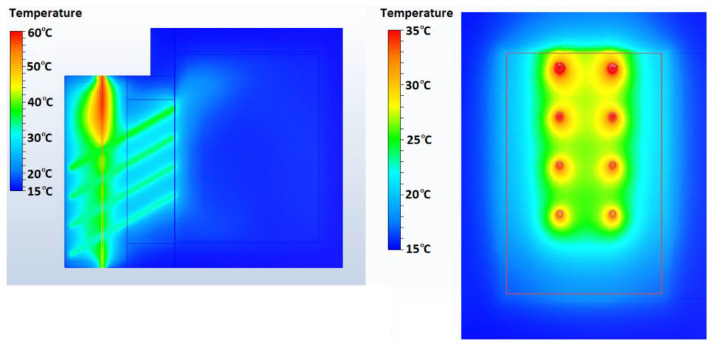
Numerical simulation for CW02—100 W/m^2^.

**Figure 12:**
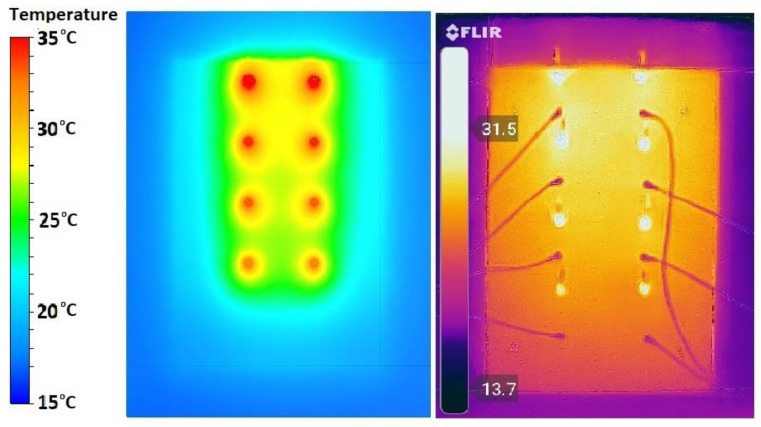
Comparison between simulation and infrared camera for CW03—250 W/m^2^.

All 4 concrete blocks were subjected to numerical simulations. Figure 11 presents a section of the global system using the CW02 concrete module, where how heat is transferred from the frontal part of the system to the room using the heat pipes inserted in the wall can be observed. The right image presents the face of the wall inside the room at the end of the charging cycle. A photograph taken with the infrared camera for CW03 with an applied heat flux of 250 W/m^2^ was compared to an image from the simulations, and the results were very similar. The comparison is presented in Figure 12.

The same boundary conditions used in the tests were applied to numerical simulations of each global system containing the four different types of concrete block. The average temperature of the external face of the wall was determined. The results are presented in Table 8.

## 4. Discussion

All 4 concrete samples showed promising results. Sample CW02, a mixture of concrete, fly ash, and chopped PET, registered the highest temperature in the experiments at the end of the charging cycle, at 28.2 °C. The numerical simulations confirmed this temperature peak.

Compared to the control sample CW01, sample CW03 behaved very similarly during the charging cycle. For all of the heat fluxes studied in the experiments, the temperature difference between the time when the lamps were switched off and the time when the discharging cycle ended was marginally smaller in CW03. That means the concrete sample with fly ash and sawdust is better at storing heat than the control sample.

Temperature differences between the concrete sample CW04 with fly ash and granular polystyrene, and CW03, were from 0.2 to 0.6 °C. However, their behavior was different during the discharging phase. CW04 had the smallest heat storage capacity, with an average temperature change of 6 °C between hours 10 and 24. A more detailed view of the temperature drop during the discharging phase is presented in Table 9. These numbers were calculated using the difference in average temperature between minute 600 and minute 1440 from the experiments.

The results recorded by the temperature sensors were confirmed by thermal imaging photos and also by numerical simulations. The temperatures resulting from the numerical simulations were slightly higher than those of the experiments, with values between 1 and 3 °C. This can be explained by the fact that the software does not take into account convective heat losses.

## 5. Conclusions

The main goal of the research was to convert solar energy from bright building façades in an efficient way, putting forth a conversion-accumulation-solar energy delivery system based on the construction of an inventive solar panel with heat pipes and a heat storage wall for creating passive buildings. This system converts the solar energy from the sunny façades into thermal energy, which is then transferred by the integrated heat pipes in a large element with high thermal inertia. The thermal energy is saved during the day, and is released at night by using insulated shutters to maintain a comfortable temperature in the room.

Four concrete samples were cast with standard aggregates and waste aggregates in the mixture: CW01—corresponds to the concrete control sample; CW02—corresponds to concrete with fly ash and chopped PET; CW03—corresponds to concrete with fly ash and sawdust; CW04—corresponds to concrete with fly ash and granular polystyrene. These were integrated into a solar recovery system. Each global system was subjected to four heat fluxes of 100 W/m^2^, 150 W/m^2^, 200 W/m^2^, and 250 W/m^2^. The results were recorded by temperature sensors, and were confirmed by thermal imaging photos and also by numerical simulations.

CW02 had the most efficient mixture, resulting in an increased efficiency of 3.3% higher on average at the end of the charging cycle than the efficiency of CW01, the control sample. CW03 was the most efficient during the discharging cycle, losing on average 5.0 compared with 5.5 °C, the average temperature drop of CW01.

The original idea of the passive, eco-friendly system used to increase the energy efficiency of buildings may be quickly and cheaply installed in modules. The system can be used in climate zones where moderate solar heat flux is present throughout the entire year since it can be applied to all buildings, old and new, meeting heat demand even with low heat fluxes applied. The heat pipe system, in addition to improving a building’s energy efficiency, has a major economic impact by lowering overall heating costs and a large environmental impact by lowering CO_2_ emissions. Also, recycling materials are included in the concrete mixture, which benefits the environment.

The final conclusion of this study is that the suggested global system is a practical and effective way to recover solar energy from sunny building façades in order to create passive structures, even in situations with low solar radiation intensities of 100 to 250 W/m^2^.

## Figures and Tables

**Table 1:** The average temperature on the surface of the wall for CW01.

Heat Flux	After 1 h	After 5 h	After 10 h
100 W/m^2^	16.0 °C	20.1 °C	23.7 °C
150 W/m^2^	16.1 °C	20.7 °C	24.5 °C
200 W/m^2^	16.2 °C	21.2 °C	24.9 °C
250 W/m^2^	16.2 °C	22.3 °C	27.2 °C

**Table 2:** The average temperature on the surface of the wall for CW02.

Heat Flux	After 1 h	After 5 h	After 10 h
100 W/m^2^	15.9 °C	20.0 °C	23.3 °C
150 W/m^2^	16.1 °C	21.3 °C	25.1 °C
200 W/m^2^	16.7 °C	22.8 °C	26.9 °C
250 W/m^2^	17.4 °C	23.8 °C	28.2 °C

**Table 3:** The average temperature on the surface of the wall for CW03.

Heat Flux	After 1 h	After 5 h	After 10 h
100 W/m^2^	15.9 °C	19.9 °C	23.3 °C
150 W/m^2^	16.0 °C	20.7 °C	24.0 °C
200 W/m^2^	16.2 °C	21.5 °C	25.1 °C
250 W/m^2^	16.3 °C	22.3 °C	26.1 °C

**Table 4:** The average temperature on the surface of the wall for CW04.

Heat Flux	After 1 h	After 5 h	After 10 h
100 W/m^2^	16.2 °C	20.3 °C	23.3 °C
150 W/m^2^	16.2 °C	20.5 °C	23.6 °C
200 W/m^2^	16.2 °C	21.4 °C	24.3 °C
250 W/m^2^	16.4 °C	22.1 °C	26.4°C

**Table 5:** Heat flux through the wall.

Heat Flux	CW01	CW02	CW03	CW04
100 W/m^2^	1174.2 W/m^2^	1143.9 W/m^2^	1139.8 W/m^2^	1149.6 W/m^2^
150 W/m^2^	1774.1 W/m^2^	1818.2 W/m^2^	1736.5 W/m^2^	1715.1 W/m^2^
200 W/m^2^	2233.3 W/m^2^	2405.3 W/m^2^	2250.0 W/m^2^	2200.0 W/m^2^
250 W/m^2^	2971.8 W/m^2^	3106.1 W/m^2^	2856.3 W/m^2^	2925.0 W/m^2^

**Table 6:** Temperature comparison between experimental and infrared camera results for CW02.

		T1	T2	T3	T4	T5	T6	T7	T8
Sensors	250 W/m^2^	30.2 °C	30.0 °C	29.2 °C	30.4 °C	28.3 °C	29.0 °C	25.4 °C	26.2 °C
IR		29.9 °C	29.8 °C	29.1 °C	30.3 °C	28.1 °C	28.9 °C	25.3 °C	26.0 °C
Sensors	200 W/m^2^	28.4 °C	29.6 °C	27.9 °C	28.5 °C	26.8 °C	27.3 °C	24.6 °C	24.9 °C
IR		28.1 °C	29.5 °C	27.7 °C	28.2 °C	26.7 °C	27.2 °C	24.6 °C	24.8 °C
Sensors	150 W/m^2^	26.6 °C	26.5 °C	26.5 °C	26.6 °C	25.0 °C	25.5 °C	23.0 °C	23.4 °C
IR		26.4 °C	26.5 °C	26.4 °C	26.4 °C	24.9 °C	25.3 °C	22.8 °C	23.3 °C
Sensors	100 W/m^2^	24.6 °C	24.2 °C	24.3 °C	24.8 °C	23.3 °C	23.8 °C	21.5 °C	22.0 °C
IR		24.3 °C	24.1 °C	24.1 °C	24.5 °C	23.1 °C	23.7 °C	21.4 °C	22.0 °C

**Table 7:** The average temperatures resulted from numerical simulations.

	CW00	CW01	CW02	CW03	CW04
Wall temp.	21.3 °C	26.8 °C	30.3 °C	24.9 °C	29.7 °C
Room temp.	19.6 °C	27.2 °C	30.6 °C	25.3 °C	29.9 °C

**Table 8:** Results of the numerical simulations.

	100 W/m^2^	150 W/m^2^	200 W/m^2^	250 W/m^2^
CW01	24.4 °C	25.9 °C	26.4 °C	28.2 °C
CW02	24.4 °C	26.8 °C	29.1 °C	30.3 °C
CW03	24.2 °C	25.6 °C	26.6 °C	27.8 °C
CW04	24.5 °C	25.3 °C	25.2 °C	27.7 °C

**Table 9:** The temperature drop during the discharging phase.

	100 W/m^2^	150 W/m^2^	200 W/m^2^	250 W/m^2^
CW01	5.0 °C	5.0 °C	6.1 °C	6.0 °C
CW02	4.3 °C	4.7 °C	5.8 °C	6.4 °C
CW03	4.1 °C	5.0 °C	5.6 °C	5.2 °C
CW04	6.1 °C	5.0 °C	6.1 °C	6.8 °C

## Data Availability

Not applicable.

## Nomenclature

CW01	Concrete control sample
CW02	Concrete with fly ash and chopped PET
CW03	Concrete with fly ash and sawdust
CW04	Concrete with fly ash and granular polystyrene
φ	Heat flux [W/m^2^]
T_x_	Thermocouple sensors
*Q*	Heat gain of the wall [*W*]
*k*	Thermal conductivity [W/mK]
*A*	Area [m^2^]
∆*T*	Temperature difference [°C]
*d*	Width [m]
